# Utilizing Electrocochleography as a Microphone for Fully Implantable Cochlear Implants

**DOI:** 10.1038/s41598-020-60694-z

**Published:** 2020-02-28

**Authors:** William Jason Riggs, Meghan M. Hiss, Jeffrey Skidmore, Varun V. Varadarajan, Jameson K. Mattingly, Aaron C. Moberly, Oliver F. Adunka

**Affiliations:** Department of Otolaryngology, Head & Neck Surgery, The Ohio State University College of Medicine, Columbus, OH USA

**Keywords:** Inner ear, Translational research

## Abstract

Current cochlear implants (CIs) are semi-implantable devices with an externally worn sound processor that hosts the microphone and sound processor. A fully implantable device, however, would ultimately be desirable as it would be of great benefit to recipients. While some prototypes have been designed and used in a few select cases, one main stumbling block is the sound input. Specifically, subdermal implantable microphone technology has been poised with physiologic issues such as sound distortion and signal attenuation under the skin. Here we propose an alternative method that utilizes a physiologic response composed of an electrical field generated by the sensory cells of the inner ear to serve as a sound source microphone for fully implantable hearing technology such as CIs. Electrophysiological results obtained from 14 participants (adult and pediatric) document the feasibility of capturing speech properties within the electrocochleography (ECochG) response. Degradation of formant properties of the stimuli /da/ and /ba/ are evaluated across various degrees of hearing loss. Preliminary results suggest proof-of-concept of using the ECochG response as a microphone is feasible to capture vital properties of speech. However, further signal processing refinement is needed in addition to utilization of an intracochlear recording location to likely improve signal fidelity.

## Introduction

To date, it is estimated that as many as 466 million individuals worldwide have hearing loss as defined as an average hearing level of ≥35 dB HL by pure-tone audiometry^1^. Treatment options for hearing loss typically depend on the severity of the hearing loss. Cochlear implants (CI) have long been a treatment option for individuals with severe-to-profound hearing loss; however, with advancements in technology, candidacy criteria have expanded to include individuals with greater amounts of residual hearing. With this trend, the focus has shifted toward developing techniques and technology to allow for the preservation of residual hearing, as this has been shown to be important in obtaining optimal outcomes through the use of electric-acoustic stimulation. That is, in patients who receive CIs but maintain some useable residual hearing, the implanted ear can be stimulated using the ipsilateral combination of electric (CI) and acoustic (hearing aid)^2,3^.

In attempts to achieve preservation of residual hearing, implementation of electrocochleography (ECochG) at the time of CI surgery has recently been described. ECochG is a tool that allows electrophysiological assessment of the peripheral auditory system (i.e., the cochlea and auditory nerve) by using acoustic stimulation. Specifically, ECochG has been used as a monitoring tool during CI surgery in an effort to provide real-time feedback of inner ear physiology that allows for modifying surgical technique in an attempt to avoid trauma caused by the electrode insertion, hence preserving residual hearing^4–6^. The use of ECochG is not new, but its use in CI surgery is novel. Specifically, new technology has recently been introduced that allows the ECochG signal to be recorded through the CI electrode array (CI-ECochG). This technique has great advantages: recording ECochG through an electrode contact of the CI provides favorable signal-to-noise ratios allowing for the acquisition of physiologic data in real-time even in ears with substantial degrees of hearing loss^5,6^. In addition to its surgical utility, authors have shown that this technique can accurately predict post-operative behavioral audiometric thresholds of residual hearing^7^.

Due to this recent technological advancement, in theory additional applications could be developed using the CI-ECochG platform. Of particular interest would be an application that allows for CI’s that are “fully implantable”. Current standard CI systems contain two main components: (1) an internal electrode array and receiver stimulator which are implanted at the time of surgery and (2) an external speech processor with a coil headpiece which connects to the internal portion via electromagnetic induction^8^. The battery source is external and connected to the speech processor that is typically worn over the ear. The speech processor contains multiple microphone configurations that detect acoustic information of the external environment. While batteries have been developed for fully implantable CIs (meaning no external components are required), a main barrier to fully implantable devices is how to capture sounds when there is no external microphone component. To address this issue, various implantable microphone concepts have been explored in the past, such as subcutaneous devices^9^ and options which obtain mechanical sound energy from the middle ear ossicular chain^10^. However, each of these options has drawbacks, including interference from body noise, substantial attenuation of sound signals, high power consumption, and difficult calibration. However, fully implantable CIs are highly desirable and of great clinical relevance to recipients, permitting around-the-clock CI use, enhanced safety due to constant access to sound input, improved cosmetics, and the ability to participate in water sport activities without special water-proof equipment.

The current experiment proposes a novel application of CI-ECochG technology, relying on the cochlear microphonic (CM) response of the inner ear, to serve as an implanted, internal microphone for future CI devices. The ECochG signal is a gross evoked potential that is dominated by cochlear hair cell activity represented by the CM and summating potentials^11,12^ as well as contributions from neural sources^13,14^. The CM is thought to predominately reflect evoked activity of hair cells (outer) due to basilar membrane motion, with its morphology alternating in polarity and following the phase of the incoming signal^15^. First described by Weaver and Bray^16^, it was termed the ‘microphone’ potential as the response typically mimics the acoustic waveform generated from an external source that is transferred from the external ear canal and middle ear. Owing to this property, the CM response could serve as an internal microphone for a hearing device such as a CI. Thus, one potential application would be to utilize this property of the CM obtained in the ECochG response to back-trace the acoustic properties of the original sound signal. That is, recording the CM response from an intracochlear electrode and subsequently processing the response (as an external CI speech processor would do) and delivering it to the internal receiver stimulator as a representation of the acoustic signal that was delivered to the ear, could support the development of an implantable microphone as a vital component of a fully implantable CI. However, while CI-ECochG platforms are clinically available, the use of this technology as a microphone is not available in current CI platforms. Thus, the current study employed an extracochlear round window (RW) approach to demonstrate proof-of–concept for this potential application in future CI technology.

In order to utilize ECochG CM responses as an internal microphone, the resulting signal’s quality would be paramount. Specifically, to utilize the CM response as a microphone that is ultimately used to drive stimulation of the CI, speech information (acoustic features/properties) must be preserved within the ECochG response. Since the CM response is dominated by hair cell activity^17^, sensorineural hearing loss (SNHL) would likely cause degradation of how well the incoming signal is represented by the CM. Therefore, one objective of this study was to assess the ability of the CM to accurately represent a speech signal in ears with SNHL.

**Figure 1:**
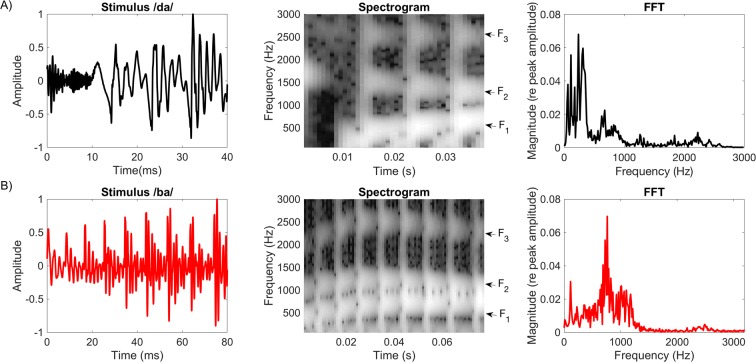
(**A**) Time and spectral domain representation of the acoustic stimulus/da/. (**B**) Time and spectral domain representation of the acoustic stimulus/ba/. The arrows illustrate the spectrogram format structures (F_1_-F_3_) for both stimuli.

An important acoustic property of a speech signal would be that of formant structure. Formants are concentrated regions of energy that represent the acoustic resonances of the vocal tract^18^. That is, the glottal folds generate a fundamental frequency (pitch – F_0_) and the resonances of the vocal tract following glottal vibration create a multi-formant structure numbered in an upward fashion (F_1_, F_2_, F_3_…) as frequency bands increase. F_0_ are important for identification of pitch of the voice while the acoustic behavior of the formants following F_0_ are critical for identification/differentiation of the speech sound^19,20^. As the main objective was to determine the feasibility of utilizing the ECochG signal as a microphone, we wanted to evaluate how representation (frequency and intensity) of the formant structure (F_1_-F_3_) behaved in various degrees of SNHL using vowel-dominated phonemes (Fig. 1A,B) when recorded from the RW. With SNHL, it is expected that both amplitudes (valley-peak) and frequency resolution are reduced due to fewer sensory hair cells and broadened auditory filters^21^. Perceptually, vowel identification has been reported to be fairly robust even in ears with significant degrees of hearing loss^22–25^. Thus, we predicted that a substantial portion of the formant structure would be encoded adequately at the level of the cochlear hair cells despite significant degrees of hearing loss.

The present report evaluated the ECochG response’s capability, when recorded from the RW of the cochlea, to represent formant structure of an acoustic speech signal in humans undergoing a variety of otologic surgeries with diverse preoperative hearing conditions. That is, both CI participants (severe hearing loss) and non-CI participants (mild-to-severe) were included to establish how well the speech signal can be represented by the ECochG and evaluate its degradation with increasing severity of hearing loss.

## Results

### Hearing profiles

**Figure 2:**
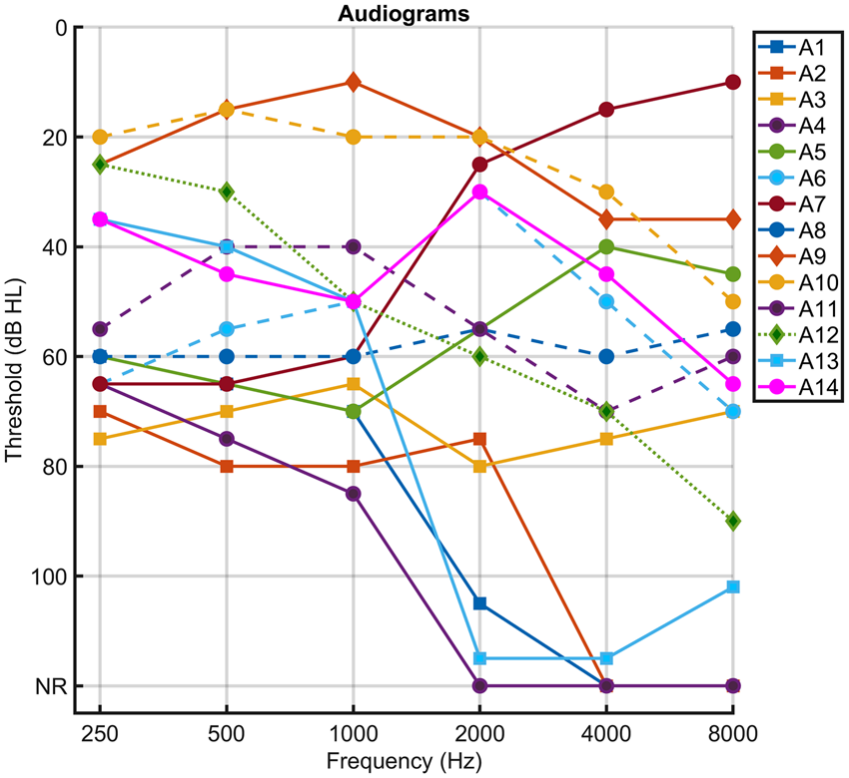
Audiometric profiles for study participants. Squares represent those who received cochlear implants, circles represent those who were diagnosed with Meniere’s disease and underwent endolymphatic sac decompression and shunt placement or labyrinthectomy, and diamonds represent those who were having a vestibular schwannoma removed. NR refers to no response at the limits of the audiometer.

Demographic and surgical information of study participants (n = 14) can be found in Table 1. Results of audiometric testing near the time of surgery can be seen in Fig. 2. The study group exhibited widespread audiometric thresholds ranging from mild to profound SNHL. Pure tone average (PTA- 0.5, 1, 2 kHz) ranged from 15–93.33 dB HL (mean: 56.21 dB, SD: 24.8 dB) with word recognition scores (WRS) ranging from 0–100% (mean: 45.45%; SD: 37.41%). Note, participant A4 was diagnosed with auditory neuropathy spectrum disorder (ANSD), previously shown to have robust cochlear function exhibited by large CM responses but neural dyssynchrony and 0% WRS.

**Table 1:** Demographic/Surgical information of subjects who participated in this study.

Subject	AAT	Ear	Recording Site	Surgery	PTA	WRS (%)
A1	76	L	RW	CI	80	28
A2	70	R	RW	CI	78	32
A3	13	L	RW	CI	72	16
A4*	29	L	RW	CI	93	0
A5	42	L	RW	ELS	63	76
A6	61	L	RW	ELS	45	60
A7	30	L	RW	ELS	50	96
A8	66	L	RW	Labyrinthectomy/CI	58	36
A9	58	L	RW	VS Resection	15	100
A10	47	L	RW	ELS	18	100
A11	65	L	RW	Labyrinthectomy	45	40
A12	68	L	RW	VS Resection	47	88
A13	84	L	RW	CI	68	28
A14	51	R	RW	ELS	42	92

R = right, L = left, AAT = age at testing (years), RW = round window, ELS = endolymphatic sac decompression and shunt, CI = cochlear implant, VS = vestibular schwannoma, WRS = word recognition score, PTA = pure tone average. *Indicates the participant who was diagnosed with auditory neuropathy spectrum disorder (ANSD).

### Electrophysiological representation of the stimulus: Time domain

**Figure 3:**
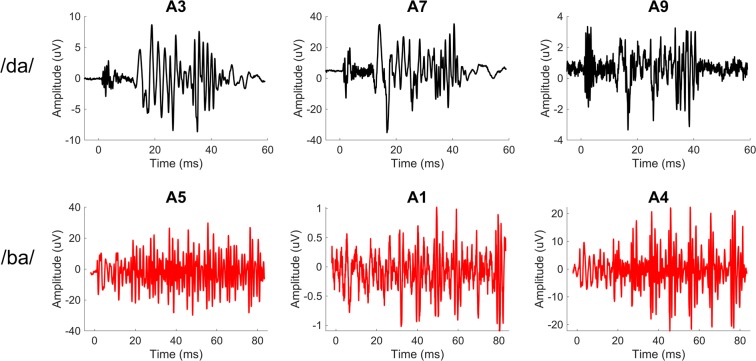
Example ECochG_diff_ responses from study participants evoked by the /da/ (**A**) and /ba/ (**B**) stimuli.

To emphasize components of the ECochG response that change with stimulus phase, such as the CM dominated portion, a difference waveform (ECochG_diff_) was created by subtracting the ECochG response evoked by the rarefaction phase from the ECochG response evoked by the condensation phase. Base-to-peak amplitudes (μV) of the non-normalized ECochG_diff_ response (time domain), measured as the region of the ECochG_diff_ response after stimulus onset that produced the maximal amplitude deflection, were calculated and for those evoked by /da/ presented at 108 dB peak equivalent sound pressure level (peSPL) ranged from 2.46–46.06 µV (n: 14, mean: 13.73 µV, SD: 13.43 µV). Amplitudes for the /ba/ responses presented at 89 dB peSPL ranged from 1.10–29.80 µV (n: 11, mean: 9.60, SD: 9.53). The difference in peaks was expected as the overall peak sound pressure level (peSPL) value for the /da/ was 19 dB louder than the /ba/. Examples of raw ECochG_diff_ responses for both stimuli can be seen in Fig. 3. In comparison to the time domain waveforms of the stimuli (Fig. 1A,B), visually, the overall fidelity (time domain representation of the stimulus) appears to be maintained in the ECochG_diff_ response. Of note, largest amplitudes were observed in participants with the diagnosis of Meniere’s disease (MD) while smallest amplitudes were typically exhibited by those receiving a CI (without MD diagnosis).

Each ECochG_diff_ response was then normalized to its peak amplitude (maximal voltage of the time domain response) for each individual participant. Following normalization, as ECochG is an evoked response, it was necessary to align (i.e. adjust in latency or lag time) the evoked ECochG_diff_ response with that of the stimulus. This was achieved with a cross-correlation approach that yielded a latency (lag time) value (ms) where the two waveforms (stimulus and ECochG response) were most highly correlated. ECochG_diff_ latency times ranged from −9.10 to −6.90 ms (mean: −7.96, SD: 0.75) for the /da/ and −6.40 to −2.90 ms (mean: −4.45, SD: 1.04) for the /ba/. Latency values were based on a single ECochG_diff_ trial for each participant and variation in lag time was expected due to the different severities of SNHL across the study group. After adjusting for lag time, Pearson product-moment correlation was ran between the stimulus and each ECochG_diff_ response. All correlations were found to be statistically significant (p < 0.05) and their coefficients can be found in Table 2. Coefficients ranged from 0.31–0.82 (mean: 0.57, SD: 0.15) and 0.35–0.83 (mean: 0.59, SD: 0.16) for the /da/ and /ba/ respectively (Table 2). Overall, this suggested a moderate to strong correlation (i.e. waveform similarity) after alignment between each ECochG_diff_ response and the stimulus based on its time domain representation for both stimuli.

**Table 2:** Evoked potential values of the difference waveform (ECochG_diff_ - subtraction of condensation and rarefaction raw waveforms) response values for stimuli /da/ and /ba/.

Sub^#^	/da/				/ba/			
	Peak Amp (µV)	Cross Correlation Lag Time (ms)	Stim-Response Correlation (r)	Structural Similarity Index (SSIM)	Peak Amp (µV)	Cross Correlation Lag Time (ms)	Stim-Response Correlation (r)	Structural Similarity Index (SSIM)
A1	3.04	−9.10	0.54*	0.18	1.10	−6.40	0.52*	0.22
A2	4.76	−8.60	0.68*	0.38	2.31	−5.80	0.35*	0.23
A3	8.65	−8.30	0.61*	0.35	2.34	−3.70	0.58*	0.26
A4	20.18	−7.40	0.31*	0.58	22.28	−2.90	0.70*	0.62
A5	46.06	−7.20	0.64*	0.52	29.80	−4.00	0.67*	0.59
A6	15.95	−7.10	0.63*	0.48	7.36	−4.20	0.42*	0.30
A7	35.17	−8.50	0.32*	0.33	16.92	−4.10	0.68*	0.53
A8	5.93	−8.60	0.60*	0.18	2.83	−4.40	0.61*	0.24
A9	3.34	−7.30	0.43*	0.47	—	—	—	—
A10	21.91	−7.10	0.45*	0.53	10.40	−4.00	0.79*	0.55
A11	4.63	−8.50	0.82*	0.39	2.34	−5.60	0.37*	0.21
A12	2.46	−8.50	0.74*	0.36	—	—	—	—
A13	2.95	−8.40	0.62*	0.26	—	—	—	—
A14	17.21	−6.90	0.60*	0.48	7.97	−3.90	0.83*	0.59

— indicates that a trial for that subject was not carried out due to timing constraints during surgery. *Indicates statistically significant (p < 0.05).

### Electrophysiological representation of the stimulus: Spectrogram

**Figure 4:**
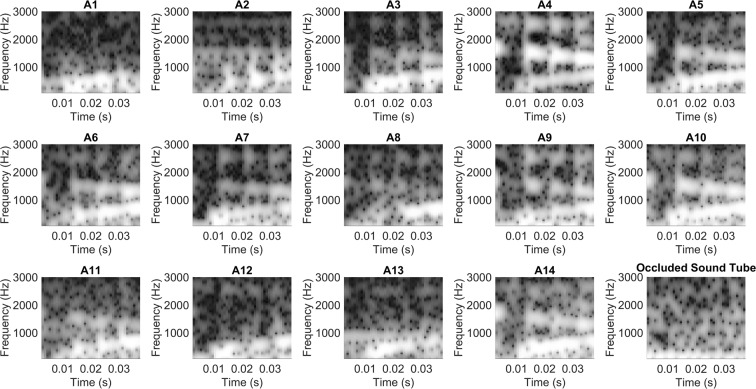
Spectrograms of the normalized ECochG_diff_ evoked by an 80 dB nHL /da/. The “Occluded Sound Tube” trial represents the average across all control trials where the sound tube was occluded with a hemostat and the stimulus presented at 80 dB nHL.

To evaluate representation of the stimulus formant frequencies over time that were present in the ECochG_diff_ response, each response was windowed into segments composed of 240 points and fast Fourier transforms (FFTs) were then used to create spectrograms of the normalized lag time aligned ECochG_diff_ responses. Spectral amplitude at the center frequency of each formant was calculated at three regions along the formant (beginning, middle, end) to determine significance above the noise floor (see Methods). If all points along each formant band were significant then this was considered full formant representation. If only one or two regions were significant per formant, then partial representation was considered. The spectrograms for each subject are shown in Fig. 4 and results of the FFT analyses indicated that the formant structure of the /da/ evoked ECochG_diff_ varied in its representation across the responses of the study group. Overall, 13 participants had full F_1_ representation present in the ECochG_diff_ response and one (A8) had a partial F_1_. Eight participants had both full F_1_ and F_2_ representation of which three (A4, A7, A9) also had full representation of all three formants while three had partial (A5, A6, A10). One participant had full F_1_ with partial F_2_ present (A11) and four participants (A1, A2, A12, A13) had only an F_1_ structure present. The averaged occluded sound tube response trial (sound tube clamped by a hemostat) can be seen in the last panel of the bottom row. Visual inspection shows minimal extraneous electrical noise with no representation of the stimulus formant structures, supporting authenticity of the evoked ECochG_diff_ responses.

**Figure 5:**
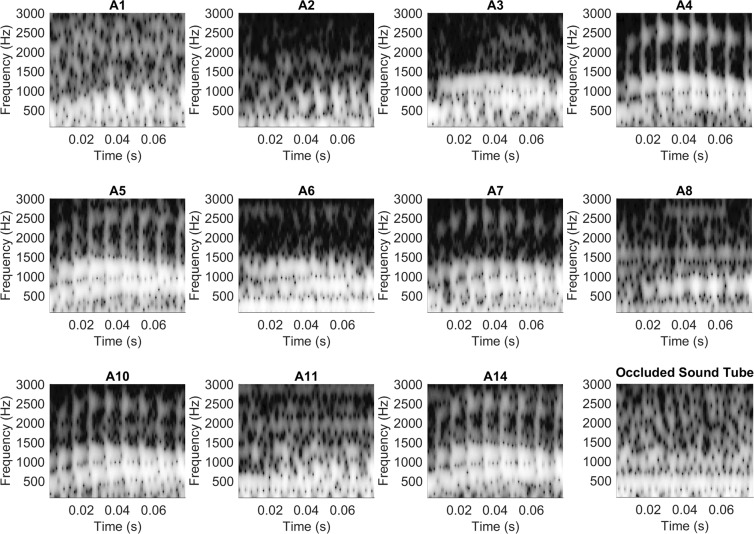
Spectrograms of the normalized ECochG_diff_ evoked by an 80 dB nHL /ba/. The “Occluded Sound Tube” trial represents the average across all control trials where the sound tube was occluded with a hemostat and the stimulus presented at 80 dB nHL.

Figure 5 displays the spectrograms for responses evoked by the /ba/ stimulus along with the averaged results of the occluded sound tube trials. Due to surgical timing constraints, A9, A12, and A13 did not have a /ba/ trial completed and were thus excluded from this analysis, thus 11 participants were included. Using the same approach as with the /da/ responses, each formant structure was measured in the same manner to determine formant representation in the response. Eight participants had full F_1_ representation while participants A1, A2 and A11 only exhibited partial representation of F_1_. Six participants had full representation of both F_1_ and F_2_, of which four (A4, A5, A10, A14) also had F_3_ present, while one had partial F_3_ (A7) and one (A3) had no measurable F_3_ response. Finally, two participants (A6, A8) had full F_1_ and only partial F_2_ representation in their ECochG_diff_ response. The final panel of the bottom row displays the average occluded sound tube trial for the /ba/ stimulus.

### Peripheral encoding of phonemic structure: Residual hearing & speech recognition

The structural similarity index (SSIM), a mathematical calculation that evaluates structure, luminance and contrast between two images to determine their overall similarity, was used for comparison of the stimulus spectrogram with the ECochG_diff_ spectrogram. This comparison revealed that spectrograms with greater formant content had the largest SSIM values. Each participant’s index value can be found in Table 2. The SSIM ranged from 0.18 to 0.58 (mean: 0.38, SD: 0.12) for the /da/ and 0.21 to 0.62 (mean: 0.37, SD: 0.17) for the /ba/. Of note, the CI participant with ANSD exhibited the highest SSIM values of the study group, which is not surprising as this condition is thought to result in normal hair cell function but poor or absent neural function. However, this finding suggested that better cochlear function is important for achieving higher SSIM values, thus we would also expect similar values in the case of a high frequency SNHL (e.g. >3 kHz).

**Figure 6:**
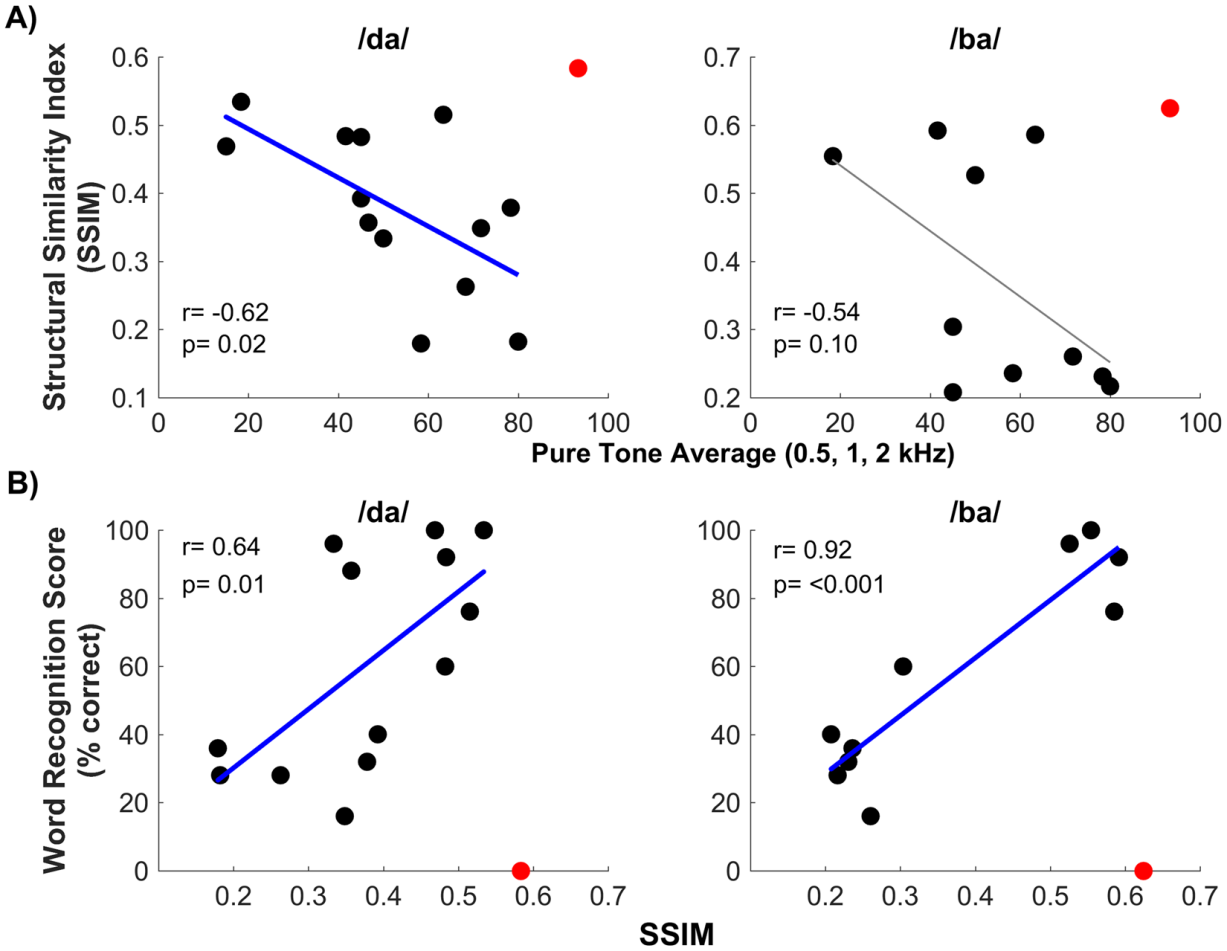
(**A**) Results of Pearson correlation between the preoperative pure tone average (PTA) and structural similarity index (SSIM). The red line indicates the line of best fit, r^2^, for the significant correlation while the grey line indicates a non-significant trend. The red dot in both plots represents the results of participant A4 who had auditory neuropathy spectrum disorder. (**B**) Results of Pearson correlation between the SSIM and speech perception testing- word recognition score (WRS- %). The red line indicates the line of best fit, r^2^ for significant correlations. As above, the red dot in both plots represents the results of participant A4 who had auditory neuropathy spectrum disorder.

To determine the influence of residual hearing on the SSIM, the pre-operative PTA was used in a Pearson product-moment correlation with the SSIM for both stimuli (Fig. 6A). A significant negative correlation was found for /da/ (n = 13, r = −0.62, p = 0.02) and a similar trend was found for /ba/ (n = 10, r = −0.54, p = 0.10) however this did not reach significance. This suggested that SSIM value was related to the amount of residual hearing as measured by the audiogram. Specifically, higher SSIM values were associated with better hearing and decreased in value as hearing worsened. Note, due to the nature of the hearing loss in ANSD, participant A4 was not included in these analyses with traditional SNHL participants as ANSD is known to result in neural dysfunction (e.g. temporal disruptions) leading to worse than expected WRS despite near normal cochlear function. However, the data for this subject are plotted in Fig. 6A (red dot) to help demonstrate the strength of the SSIM when considering traditional SNHL.

Furthermore, to determine the relevance of the formant structure contained in the ECochG_diff_ response to auditory processing when evaluated using the SSIM, Pearson product-moment correlations were ran between SSIM values and the behavioral performance score on the pre-operative speech perception task (e.g. WRS-%). This correlation was chosen as we would expect formant representation in the ECochG_diff_ response to reflect a participant’s access to spectral components of the input signal that would be important for speech perception. Indeed, the SSIM was found to positively correlate with WRS for both stimuli (/da/: n = 13, r = 0.64, p = 0.01; /ba/: n = 10, r = 0.92, p < 0.001) (Fig. 6B). As mentioned above A4 was excluded from correlations but plotted for illustrative purposes in Fig. 6B (red dot). Overall, participants with the most residual preoperative hearing typically had higher SSIM values which correlated to the their word recognition capabilities.

## Discussion

In the present paper we demonstrate our preliminary experiences utilizing the acoustically evoked ECochG response of the inner ear as a microphone source for representing speech properties of the input sound in a group of participants with SNHL. Participants with the greatest amount of pre-operative residual hearing (e.g. mild-to-moderate) exhibited the best frequency representation of the group to both stimuli (highest SSIM values). When considering those participants with hearing thresholds in the severe-to-profound range, most participants exhibited all of the F_1_ structure and often a portion of the F_2_ component as well. The proportion of formant representation in the ECochG_diff_ response (as measured by the SSIM) was significantly related to speech recognition capabilities.

### Hearing status and signal representation

Typically, SNHL involves a process whereby sensory cells (outer and inner hair cells) of the cochlea are damaged and subsequently missing, leaving few sensory receptors to detect and carry out mechano-transduction and neural activation^26^. Thus, using sensory cells as an internal microphone is limited to the extent of remaining hair cell presence in the inner ear. However, previous extra and intra-cochlear ECochG recordings have been carried out by numerous authors in recent years in instances of severe-to-profound SNHL of which many report their ability to record ECochG activity that is thought to predominantly represent the CM^4,6,7,27–30^. The challenge then becomes how well the residual sensory cells can represent the incoming speech signal, and what proportion of the acoustic properties (e.g., formant structure) is necessary to be preserved for computer algorithms to accurately identify and differentiate between speech phonemes so that the appropriate signal can be delivered to the stimulating electrode array. Here we demonstrate that despite extensive degrees of hearing loss, formant structure can be maintained to varying degrees often with at least F_1_ preserved. Thus, at a minimum, it appears that simple sound detection (signal on/off) is feasible but higher signal identification would be a greater challenge if using this technique for speech recognition. For optimal results, applications of this technology could be ideal for CI recipients who have significant residual hearing following CI surgery, as those recipients would be most likely to maintain high-fidelity speech signals from CM responses. Additionally, while around-the-clock use of this technology may not be superior to traditional microphones in terms of speech recognition, this technique would provide recipients with the option to remove the external speech processor while not completely sacrificing sound awareness.

### Implications for using the biological ECochG response as a microphone

While technology for development of fully-implantable CIs has been of growing interest, this is the first report of a technique that uses a biological response as the microphone. Other techniques have been focused on using more traditional mechanical microphones such as the electret microphone^9^. Yip *et al*. described a proof-of-concept for a fully implantable approach using a piezoelectric middle-ear sensor in a human cadaver ear whereby the sensor output obtained from the middle ear chain is used as the sound source^10^. However, due to stability issues of placement on the middle ear ossicles, carrying this out *in-vivo* is a challenging prospect. Additionally, Zhao and colleagues were able to demonstrate the feasibility of designing and using an intracochlear location of a piezoelectric transducer (micro-electro-mechanical systems xylophone) in a guinea pig model^31^. Here there is a probe that courses within the cochlea and is composed of a xylophone-like structure that is designed to resonate at different frequencies in attempts to mimic the fluid dynamics of the inner ear/ basilar membrane. However, the practical aspects of an additional intracochlear structure besides the electrode would need to be addressed.

The advantage of the current study is that no additional microphones would be necessary. That is, electrode arrays of CIs have several electrode contacts. Previous work has demonstrated the feasibility of recording acoustically evoked responses from the electrode array in implanted ears^4,5,7^. Since these studies have shown that the maximal amplitude of the ECochG response is often found at the apical reaches of the electrode array, designating this electrode location as a constant ECochG microphone while leaving the remaining electrodes of the array to electrically stimulate the auditory nerve would not require any alteration to the normal CI surgical process or CI design.

### Peripheral encoding of phonemes- *Importance for speech understanding*

The current assessment of signal representation used the SSIM, which is often employed in the visual sciences to compare images (reference and target). Our rationale for utilizing this approach was that we sought a single metric that could quantify overall fidelity/structure over time of the evoked response compared to the input acoustic signal. Its use here is novel and yielded interesting clinical relevance. First, the amount of residual hearing, as measured by preoperative audiometry, was correlated with the SSIM. This was true for the /da/ responses and while this same trend existed for responses to /ba/, this correlation did not reach statistical significance. We assume the smaller number of subjects available for the /ba/ correlation likely impacted this outcome. Regardless, these findings suggested that SSIM value was related to the amount of residual hearing of the participant.

Secondly, the amount of formant structure of the stimulus signal that was represented in the ECochG_diff_ response, as measured by the SSIM, strongly correlated to the participant’s perceptual ability to understand speech as measured by a monosyllabic word list (NU-6). This is somewhat intuitive since SNHL is thought to result in a reduced number of hair cells and a subsequent broadening of auditory filters of the cochlea and thus reduced audibility and frequency resolution^32–34^. However, the phoneme-evoked response helps demonstrate the importance of audibility and frequency selectivity by the ear at the peripheral level and its relation to speech recognition. That is, the spectral analyses of the /da/ and /ba/ evoked responses covered nearly ¾ of the speech spectrum (bandwidth ranging from ~100 Hz to ~2500 Hz). Therefore, in the event that there were sensory hair cells in this frequency range (through ~2500 Hz) remaining that were able to accurately encode all three formants, we would expect that this spectral reach would be similar across other phonemes. We attribute this finding to similar mechanisms which underlie the speech intelligibility index (SII)^35,36^. The basis of the SII is that the greater the residual hearing remaining to encode frequencies across the speech spectrum, the better the WRS, as long as the sound is presented at an audible level. Here we see that at a loud level, WRS is predicted by the proportion of spectral encoding across most of the speech frequency bands as measured in the phoneme evoked ECochG_diff_ response. Thus, the greater proportion of the speech spectrum that is available to the participant, the better the ability to recognize speech.

### Improvements and limitations

Future development of this technique would be greatly refined by using an intracochlear recording electrode, ideally with an apical location. For proof-of-concept, the current study uses an extracochlear recording location to explore the concept of a biological microphone. Previous studies have shown that when recording ECochG intracochlearly, the response can be as much as three times larger than when recording at an extrocochlear location such as the RW^37^. Hence, we would expect improved signal representation using such a technique. Note, anecdotally when the ECochG_diff_ responses were reconstructed as audio files and played audibly, many of the responses were intelligible to the authors.

While the current study’s objective was to demonstrate feasibility of using the ECochG signal to recreate the incoming speech signal, multiple study limitations exist. ECochG is an evoked potential that often requires multiple averages. Thus, the response without averaging would need to be evaluated for its utility for speech representation. Consequently, ears with greater residual hearing are likely required to attain appropriate signal-to-noise ratios. Additionally, we employed two stimuli that had relatively intense peak amplitude values. Phonemes are complex acoustic signals that contain both regions of soft and intense vocalizations. Thus, in addition to using an intracochlear electrode contact as the active recording site, evaluating ECochG representation to stimuli that have less overall peak intensity levels would be of clinical relevance. Finally, due to the constraints of our evoked potential equipment (lower sampling rate with longer duration stimuli), our ECochG recordings were limited to the upper frequency region of 3 kHz. As this upper limit does not encompass the entire speech spectrum, we were unable to evaluate how higher formant structures that are important for speech understanding would be represented in the ECochG response.

## Conclusion

Here we demonstrate the feasibility of utilizing ECochG as a microphone in ears with varying severities of hearing loss. Overall the ECochG_diff_ response exhibited modest replicability of the stimulus spectrum when residual hearing was in the mild-to-moderate range and expectedly decreased in replicability as hearing loss worsened. The similarity between the ECochG response and the stimulus (as measured by the SSIM) significantly correlating with WRS signified the importance of peripheral encoding to speech perception capability.

## Methods

This study included 14 participants (13 adults [≥18 years] and one pediatric) undergoing various otologic/neurotologic procedures. The average age at the time of testing ranged from 13–76 years (mean 50.6 yrs, SD: 20.1 yrs). Study approval was obtained through the Institutional Review Board of the Ohio State University and all experiments were performed in accordance with relevant guidelines and regulations. All adult participants provided verbal and written informed consent prior to participation and written parental informed consent and participant verbal assent were obtained for anyone under 18 years of age prior to participation.

### Audiometry

As part of the standard clinical protocol at the study institution, all participants underwent a comprehensive audiometric evaluation by a licensed audiologist using a modified Hughson-Westlake procedure^38^ prior to surgery. Speech recognition ability was evaluated using the Northwestern University Auditory Test No. 6 (NU-6)^39^, a monosyllabic word test with a consonant-nucleus-consonant construction, presented at suprathreshold levels. Audiometric thresholds, PTA, and WRS (% correct) were obtained via chart review.

### Acoustic stimuli

Target stimuli for electrophysiological testing were two synthesized (Klatt software- SenSyn, Sensimetrics Corporation, Malden, MA) consonant vowel stop bursts (48 kHz sampling rate), a 40 ms /da/ and an 80 ms /ba/, presented in alternating polarity (rarefaction/condensation). Each stimulus phase was presented for 250 repetitions for a total of 500 repetitions. These stimuli were chosen due to their established use in previous studies using complex auditory brainstem responses^40–43^. Both stimuli were composed of dynamic aspects (frequency-varying). The /da/ contained initial aharmonic energy components and broadband frication which is immediately followed by a spectrally dynamic formant transition to the vowel which dominates approximately ¾ of the signal^43^. The spectrum of the /da/ consisted of a rising fundamental (F_0_ [103–125 Hz]) with three formants (F_1_, F_2_, F_3_) which vary over time from 220 to 720 Hz (F_1_), 1700 to 1240 Hz (F_2_), and 2580 to 2500 (F_3_) over the last 30 ms of the signal. The spectrum of the /ba/ was composed of an F_0_ at 120 Hz and three formants varying over time: F_1_ (400 Hz-750 Hz), F_2_ (1000 Hz-1200 Hz), and F_3_ (2000–2500 kHz). Figure 1 portrays both stimuli in their time domains and their corresponding spectral domains. Stimulation levels were calibrated in units of dB peSPL using a 1 inch 2 cc coupler routed to a sound level meter (System 824, Larson Davis, Depew, NY). The /da/ stimulus was presented at 108 dB peSPL while the /ba/ was presented at 89 dB peSPL. The difference in intensity was due to our interest in assessing how the ECochG response could represent multiple phonemes as well as to assess degradation caused by lower intensity levels. However, due to time constraints of performing the electrophysiological recordings during surgery, we were limited in time available for data acquisition, thus we arbitrarily chose to employ two stimuli with different intensities for establishing proof-of-concept.

### Surgical and electrocochleography recording set-up

ECochG recordings were obtained for all participants intraoperatively at the time of surgical intervention. Intraoperatively, a mastoidectomy was performed followed by a facial recess approach for all procedures (endolymphatic sac decompression and shunt [ELS], labyrinthectomy, and CI). Prior to endolymphatic sac opening (during ELS), labyrinthectomy drilling, or prior to RW opening/electrode insertion (CI surgery), a monopolar probe (Kartush raspatory probe, Plainsboro, NJ) was positioned at the RW niche. The RW was always intact for the ECochG recordings and prior to any surgical intervention to the cochlea or vestibular structures. The evoked signal was recorded differentially from the RW probe to a reference electrode placed at the contralateral mastoid (Mc) and a ground (Neuroline 720, Ambu Inc, Ballerup, Denmark) placed at the forehead (Fz). Stimulus delivery and recording of electrophysiological responses were controlled using a Bio-logic Navigator Pro (Natus Medical Inc., San Carlos, CA) evoked potential system. Stimuli were delivered through a transducer (ER-3, Etymōtic Research, Elk Grove Village, IL) connected to a sound tube to a foam insert earphone placed in the external auditory canal. The high-pass filter was set at 70 Hz and low-pass was at 3000 Hz. Due to the recording epoch of the evoked potential equipment being fixed at 1024 points and different stimuli durations (/da/ :40 ms; /ba/: 80 ms), each /da/ trial was sampled at 16 kHz and each /ba/ trial was sampled at 12 kHz. Signals were amplified at 50,000x with artifact rejection level set at 47.5 µV. Each trial was typically followed with an occluded sound tube run (control trial) where a hemostat was placed across the sound tube blocking acoustic delivery to the ear canal, visually allowing for detection of electromagnetic contamination.

### Electrophysiological analysis

ECochG results were processed off-line and analyzed using MATLAB R2019a (MathWorks Corp., Natlick, MA) with custom software procedures. As our objective was to evaluate the CM’s representation of the speech-like stimulus signal, the condensation and rarefaction traces were extracted and used to calculate a difference curve (condensation – rarefaction = *ECochG*_*diff*_). This calculated waveform, while not perfect at eliminating the neural portion, helped emphasize the CM response and stimulus formant structure while minimizing neural contributions from the onset CAP^7,43–45^. After calculating the ECochG_diff_ curve, maximal amplitude defined as base-to-peak amplitude (μV) of the non-normalized ECochG_diff_ response (time domain) measured as the point of the ECochG_diff_ response after stimulus onset that produced the maximal amplitude deflection was calculated for each participant. Subsequently, each ECochG_diff_ response was then normalized to its peak amplitude.

### Stimulus-to-response correlation (Amplitude alignment)

Correlation analysis was performed to quantify how well the stimulus was represented by the ECochG_diff_ response. First, in order to align the two waveforms, the ECochG_dtff_ response was up-sampled to the sampling frequency of the stimulus and then shifted in time. The time shift was found by performing cross-correlation and was the lag time or latency (ms) corresponding to the point of highest correlation between the waveforms. Cross-correlation slides the ECochG_diff_ response (which has a longer recording window than the stimulus duration) along the x-axis of the stimulus (time domain) and calculates the integral of the stimulus and ECochG’s product at each sampled point^46^. The point at which this calculation is maximized becomes the point of alignment between the stimulus and ECochG_diff_ response. Thus, the ECochG_diff_ response is then shifted according to the latency. After alignment of the signals, the ECochG_diff_ response was windowed from 0–40 ms (same time scale as the /da/ stimulus) or 0–80 ms (same time scale as the /ba/ stimulus). Finally, Pearson product-moment correlation (r) between the two waveforms was calculated and description of correlation strength (e.g. “moderate”) was based on Cohen’s *r* classification system^47^. This approach established similarity between waveform morphology of ECochG_diff_ and stimulus within the time-domain. For Pearson correlations, all tests were two tailed and statistical significance was determined at the 95% confidence level.

### Spectrogram and structural similarity index (SSIM)

After time-domain alignment the ECochG_diff_ response was analyzed in its frequency domain using spectrogram analysis to evaluate spectro-temporal aspects (frequency variations over time). Spectrograms contained time segments composed of 240 points each that were each shaped by a Hamming window, were broad-band with a window length of 0.005 seconds (helped emphasize formant structure rather than pitch (F_0_) structure), had a frequency step of 10 Hz, were displayed with a view range of 70 Hz- 3000 Hz (same as ECochG filter settings), and were then gray-scaled (intensity range of 0–1). Frequency content for each portion of the spectrogram was calculated using FFTs with zero padding on each windowed time segment. To descriptively classify whether full or partial formant structure was present, we estimated the noise floor three bins above and below the boundary of the formant frequency of interest for three regions along the entire formant (beginning, middle, end) which was 18, 25, and 35 ms for the /da/ and 12, 40, and 68 ms for the /ba/. If all three regions were each three standard deviations above the noise floor (measured from three bins above and below the region of interest), then full formant representation was considered preserved. If only one or two of these regions were significant, then the formant structure was classified as partially present. Additionally, an occluded sound trial was conducted to confirm authenticity of the ECochG_diff_ response whereby the response was visually inspected for evidence of electromagnetic contamination (speaker artifact resembling the stimulus signal) of which no trial was found to contain this artifact.

Furthermore, as our interest was in determining how well the biological ECochG response could serve as a microphone, it was necessary to compare the frequency spectrum of the ECochG_diff_ response to that of the complex stimulus signal. For that we chose to use the SSIM to evaluate the spectra between the ECochG_diff_ response and stimulus. As formant structure is critical for differentiation of phonemes (/da/ vs /ba/), we wanted a technique that was sensitive to structural preservation (i.e. quantity and quality). The SSIM is a technique designed to evaluate two images (e.g. spectrograms), a reference (e.g. stimulus) and an image of interest (e.g. ECochG_diff_), and determine the overall similarity (distortion/error) of the two images by calculating a single overall similarity value (index)^48,49^. SSIM indices range from −1 to 1 where 1 indicates complete structural similarity (only achievable when two images are identical), 0 represents no similarity, and −1 being an exact opposite. Its value is the output of three computations between the signal spectrogram and ECochG_diff_ spectrogram: (1) linear correlation of the two signals, (2) mean luminance and (3) mean contrast. This index value was then used in separate correlations (Pearson) with PTA and WRS to evaluate clinical relevance of formant structure representation in the ECochG_diff_ response. Linear regression (least-squares) was then used to determine a line of best fit for each correlation. All statistical tests were two-tailed with significance determined at the 95% confidence level.

## Data Availability

The Matlab functions used in this study are available upon reasonable request from the corresponding author.
